# Comparative Evaluation of Chemical Profiles of Pyrrosiae Folium Originating from Three *Pyrrosia* Species by HPLC-DAD Combined with Multivariate Statistical Analysis

**DOI:** 10.3390/molecules22122122

**Published:** 2017-12-01

**Authors:** Wei Xiao, Yude Peng, Zhexu Tan, Qiuyue Lv, Chi-on Chan, Jingyu Yang, Sibao Chen

**Affiliations:** 1 Institute of Medicinal Plant Development, Chinese Academy of Medical Sciences and Peking Union Medical College, Beijing 100193, China; wxiao@implad.ac.cn (W.X.); tftzx1012@126.com (Z.T.); 18813122696@163.com (Q.L.); 2 Department of Conservation Center of Medicinal Plants, Guangxi Key Laboratory of Medicinal Resources Conservation and Genetic Improvement, Guangxi Botanical Garden of Medicinal Plants, Nanning 530023, China; pengyude@126.com; 3 State Key Laboratory of Chinese Medicine and Molecular Pharmacology (Incubation), The Hong Kong Polytechnic University Shenzhen Research Institute, Shenzhen 518057, China; on.chan@polyu.edu.hk; 4 Shenzhen Pharmcists Association, Shenzhen 518024, China; szpa2012@126.com

**Keywords:** Pyrrosiae Folium, HPLC-DAD, *Pyrrosia*, multivariate statistical analysis, principal component analysis, partial least squares discriminant analysis

## Abstract

Pyrrosiae Folium (PF) is a commonly used Chinese herb medicine originating from three *Pyrrosia* species for the treatment of urinary infection and urolithiasis. According to Chinese medicine practice, different specie origins led to some variations in the therapeutic effects of PF. To ensure the safety and efficacy of PF in clinical practice, it is necessary to establish a reliable and integrative method to distinguish PF occurring from the three species. In the present paper, a HPLC–DAD method was developed and applied to simultaneously analyze five major compounds in PF. Afterwards, multivariate statistical analyses including principal component analysis (PCA) and partial least squares discriminant analysis (PLS-DA) were applied for specie discrimination and integrative quality evaluation based on quantitative data. The chemical determination and pattern recognition results of 35 batches of PF samples indicated that PF samples from three species showed different chemical profiles and could be discriminated clearly. In conclusion, the present method is rapid and reliable for the quality assessment and species discrimination of PF.

## 1. Introduction

Pyrrosiae Folium (PF) (called “Shiwei” in Chinese) is a commonly used Chinese herbal medicine originating from the aerial part of several *Pyrrosia* plants (Polypodiaceae). PF has been used for a long time in Chinese medicine practice for the treatment of urinary infection, urolithiasis, hematuria, abnormal uterine bleeding, cough and asthma caused by damp heat, and phlegm in the lung [1]. Previous pharmacological investigations revealed that PF exhibited a number of bioactivities including antioxidant [2,3] and antibacterial [4] activities, alleviating bradyrhythmia [5], and inhibiting the formation of urinary calculi [6].

**Figure 1:**
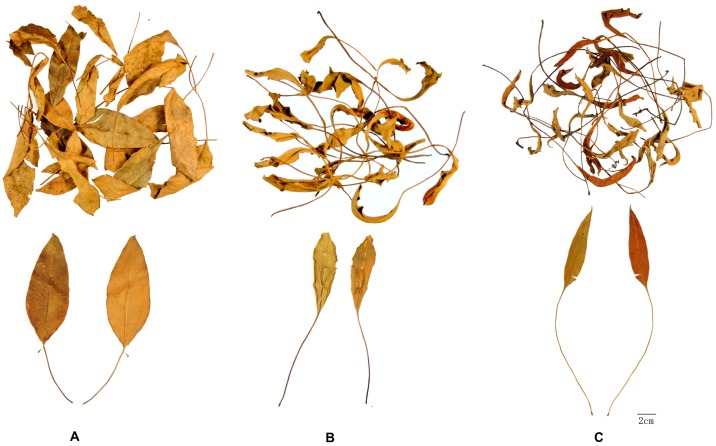
Photograph of Pyrrosiae Folium (**A**) *Pyrrosia sheareri*; (**B**) *P. lingua*; (**C**) *P. petiolosa*. (Upper: whole herb, Lower: unfolded leaf).

Owing to its pharmacological activities and therapeutic effects against a variety of disorders, PF has been widely used as main ingredient of healthy foods and herbal remedies for a long time in China. *Pyrrosia sheareri* (Bak.) Ching, *P. lingua* (Thunb.) Farwell, and *P. petiolosa* (Christ) Ching were recorded in *Chinese Pharmacopeia* as the plant origins of PF [1]. However, some practical differences in either morphology or pharmacological activity have been shown among PF originating from three plants. Sometimes PF originating from *P. sheareri* is traditionally called “Great Shiwei” due to its relatively larger leaves, and it is mainly used to treat trachitis in clinical practice, while those originating from *P. lingua* or *P. petiolosa* are referred to as “Little Shiwei” due to their relatively smaller leaves and are used to treat nephritis [7] (Figure 1). Hence, in order to guarantee its safety and efficacy in clinical practice, it is necessary to establish a feasible and integrative method to distinguish PF samples from various plant sources.

**Figure 2:**
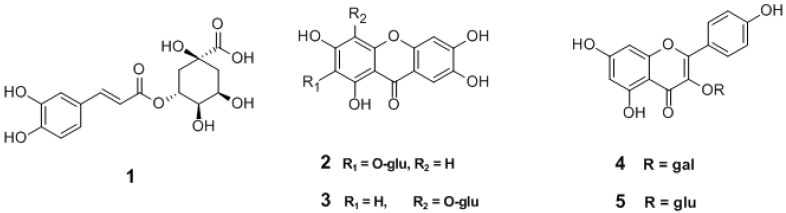
Chemical structures of compounds **1**–**5** (**1**. chlorogenic acid; **2**. mangiferin; **3**. isomangiferin; **4**. trifolin; **5**. astragalin).

Chemically, PF mainly contains essential oil, phenol, and alkaloids, etc.; among them, chlorogenic acid was selected as chemical marker for quality control by *Chinese Pharmacopeia* [1] due to its remarkable pharmacological activities including anticancer [8], antioxidant [9], and antibacterial activities [10], as well as its protection of endothelial cells [11], and metabolic modulation of glucose and fat [12]. In addition, flavonoids, such as mangiferin, isomangiferin, trifolin, and astragalin have attracted increasing attention and have been frequently used as index components in the quality assessment of PF. Previous references usually used individual or several index components as markers in the quality evaluation of PF, which then was demonstrated to be insufficient for the integrative quality assessment of PF [13,14,15]. In the present study, an HPLC-DAD method was developed and validated for the simultaneous determination of five main components, chlorogenic acid (**1**), mangiferin (**2**), isomangiferin (**3**), astragalin (**4**), and trifolin (**5**) (Figure 2) in PF samples. Furthermore, principal component analysis (PCA) and partial least squares discriminant analysis (PLS-DA) were applied to correlate and discriminate samples of three species based on a quantitative data of the analytes.

## 2. Results

### 2.1. Optimization of Extraction Conditions

**Figure 3:**
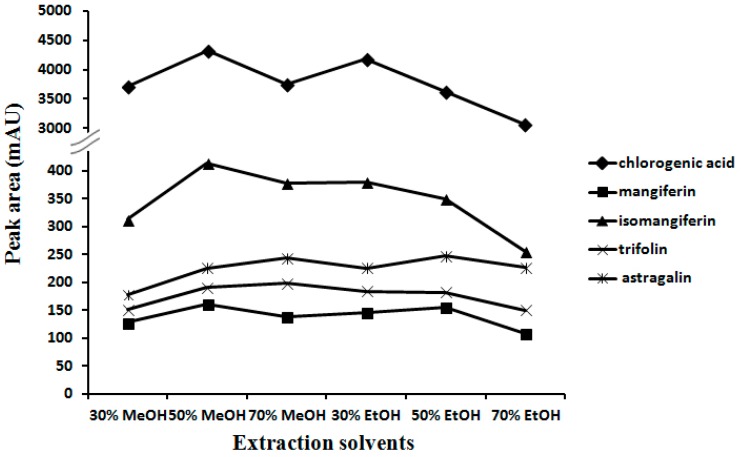
Optimization of extraction solvents.

In order to extract the five analytes from PF most effectively, extraction conditions in terms of extraction methods (refluxing, sonication, and maceration), extraction solvents (methanol and ethanol with concentrations of 70%, 50% and 30%, respectively), and extraction time (10, 30, 60, 90, 120 min) were investigated. The peak areas responding to five analytes determined in the PF sample (Batch 1) in HPLC chromatograms were recorded as a criterion for the evaluation of the efficiency of each extraction. Results indicated that sonication was the most efficient and simple procedure. As shown in Figure 3, 50% methanol showed the best extraction efficacy as it achieved the larger peak areas of the five analytes. Meanwhile, extraction for 30 min was proved to be adequate to completely extract five analytes from sample. Thus, the optimized extraction procedure was found to be sonication with 10 mL 50% methanol for 30 min.

### 2.2. Optimization of Chromatographic Conditions

**Figure 4:**
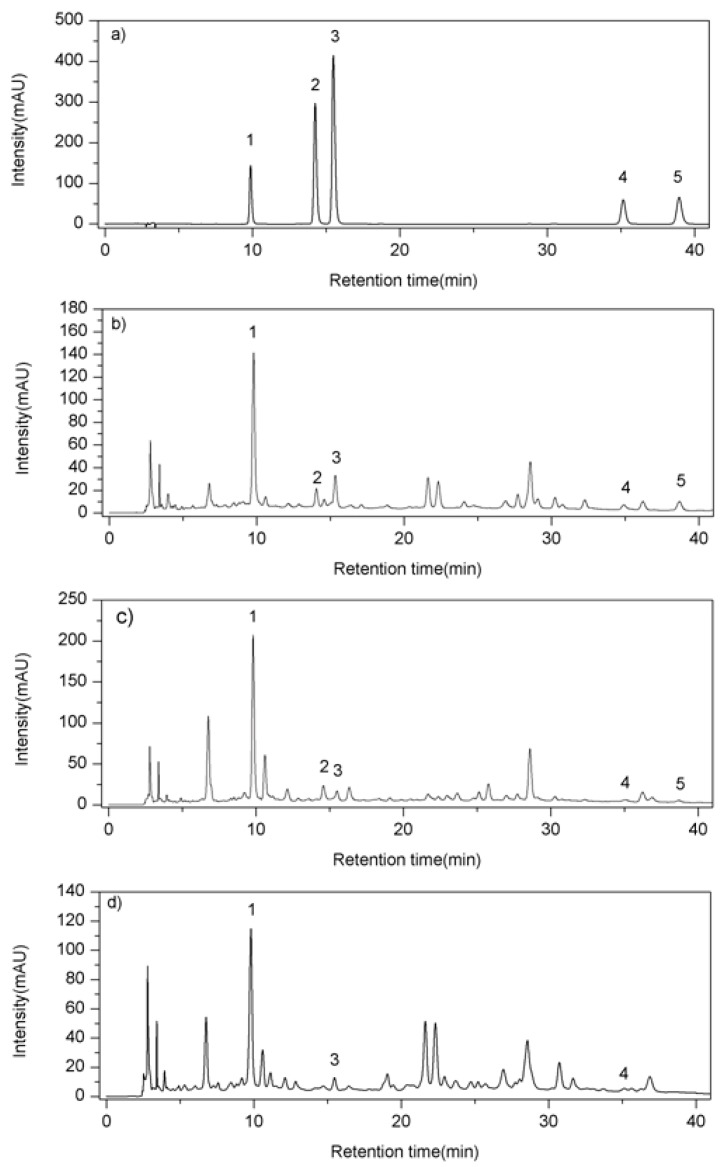
HPLC chromatograms of (**a**) standard mixture; (**b**) *Pyrrosiasheareri*; (**c**) *P. lingua*; (**d**) *P. petiolosa*. (**1**. chlorogenic acid; **2**. mangiferin; **3**. isomangiferin; **4**. trifolin; **5**. astragalin).

According to the UV spectra seized by the DAD detector, the detection wavelength was set at 254 nm, under which the five compounds showed maxima UV absorption. For mobile phase selection, various mobile phases consisting of methanol or acetonitrile combined with water or water containing different ratios of acid were investigated and compared to achieve better chromatographic separation. As a result, acetonitrile (A)-0.05% formic acid (B) was chosen as the eluting solvent system, with a gradient elution profile as follows: 9–12% of A at 0–5 min, 12–14% of A at 5–15 min, 14–17% of A at 15–25 min, 17–18% of A at 25–40 min. This method was suitable for quantitative determination as the tailing factor was less than 1.3, the resolution was more than 1.5, and the theoretical column plate numbers were more than 10,000 for each peak of analytes. The typical HPLC chromatograms of standard mixture and PF extracts of different origins are shown in Figure 4. The peaks of the tested samples were identified by comparing their retention times and UV spectra recorded by the DAD detector to those of each reference standard.

### 2.3. Method Validation

The developed method was validated in terms of linearity, precision, accuracy, and stability. As shown in Table 1, all five calibration curves exhibited good linear regressions (*r*^2^ > 0.999) within the test ranges; the limits of detection (LODs) and the limits of quantification (LOQs) of five analytes were observed to range from 0.014 to 0.085 μg/mL and from 0.047 to 0.284 μg/mL, respectively. The intra-day and inter-day variations (RSD) were less than 3% for all analytes (Table 2), which indicated that this method had excellent precision. Table 3 shows that the developed method had good accuracy, indicated by the recovery within 93% to 104% with an RSD less than 5% for all analytes. For stability test, the mixture stock solution and sample solution were analyzed every 12 h in 3 days at the room temperature. As the results shown in Table 4 display, the analytes were found to be stable within 3 days with an RSD less than 3%.

### 2.4. Sample Analysis

The proposed HPLC-DAD method was applied to analyze 35 PF samples of three species from different geographical locations. The results shown in Table 5 show that the contents of compounds **1**–**5** varied significantly due to plant origin as well as geographical location. Chlorogenic acid (**1**) is the dominant ingredient in PF and was detectable in all samples. All five analytes could be detected in *P. sheareri*, while **5** failed to be detected in *P. petiolosa*. Meanwhile, **2** and **3** were richer in *P. sheareri* than in *P. lingua* or *P. petiolosa*; conversely, the content of **4** in both *P. lingua* or *P. petiolosa* was much higher than that in *P. sheareri*. Additionally, significant variations of contents of analytes also could be observed among samples from the same plant species but growing in different geographic areas. For instance, the contents of **2** and **3** in *P. sheareri* samples varied greatly from 170.13 to 5400.74 μg/g and 421.61 to 10,916.67 μg/g, respectively, which was equivalent to approximate 30-fold and 20-fold variance among PF samples from different geographic origins.

### 2.5. Multivariate Statistical Analysis

**Figure 5:**
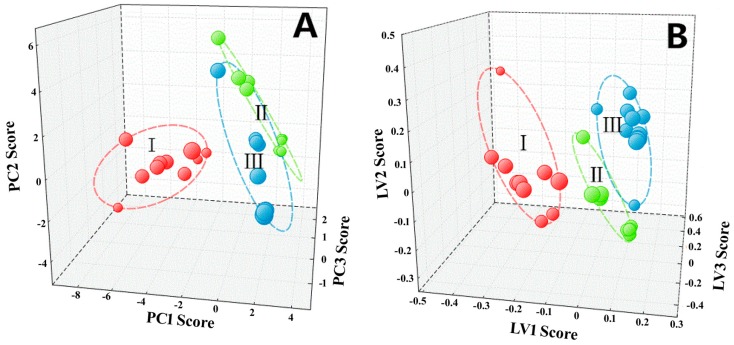
PCA score plots and PLS-DA of Pyrrosiae Folium by HPLC (**A**) PCA; (**B**) PLS-DA. (**I**: *Pyrrosia sheareri*; **II**: *P. lingua*; **III**: *P. petiolosa*).

Quantitative data acquired from HPLC analysis were subjective to PCA and PLS-DA to explore and visualize correlation and discrimination among 35 PF samples representing three plant species and different geographical areas. As shown in Figure 5A, the PCA scores plot could significantly group the tested samples into three clusters; clusters I, II, and III consisted of samples of *P. sheareri*, *P. lingua*, and *P. petiolosa*, respectively. PCA results also displayed that *P. sheareri* samples (cluster I) were distinctly different from *P. lingua* (cluster II) and *P. petiolosa* samples (cluster III), while the variance between *P. lingua* (cluster II) and *P. petiolosa* samples (cluster III) was relatively small. Similar results were observed in PLS-DA, revealing that *P. sheareri* (cluster I), *P. lingua* (cluster II), and *P. petiolosa* (cluster III) could be separated into distinct clusters as displayed in Figure 5B. Therefore, the established methods were successfully applied to the rapid identification of PF with different plant origins.

## 3. Discussion

Pyrrosiae Folium (PF) originates from three species of *Pyrrosia* genus. However, previous clinic practice revealed that PF from different plant sources showed different therapeutic effects [7], creating an urgent demand for the establishment of a comparative and concurrent method to distinguish between PF samples originating from the three species. The quality control of herbal materials is rather challenging due to its chemical complexity as well as the fact that a single active substance is not responsible for the overall pharmacological potency. Key chemical composition analysis combined with pattern recognition analysis has been demonstrated to provide information on the overall chemical composition and to distinguish different species. Previously, a few researchers quantified the amount of several or individual chemical markers, which is beneficial for the quality assessment of PF but not adequate for species discrimination [13,14,15]. According to previous study, it is clear that chlorogenic acid (**1**), mangiferin (**2**), isomangiferin (**3**), astragalin (**4**), and trifolin (**5**) are the main components of PF. Accordingly, we established a comparative method to discriminate the three species *P. sheareri*, *P. lingua* and *P. petiolosa* by simultaneously quantifying compounds **1**–**5** in PF using HPLC-DAD coupled with multivariate analysis. This method was fully validated for linearity, accuracy, precision, and recovery. Results indicated that this method is rapid, precise, and accurate. This method was further employed to determine the contents of compounds **1**–**5** in 35 PF samples originating from three species. Results revealed that there are significant variations among the three species in terms of the chemical profiles and contents of compounds **1**–**5**. In summary, chlorogenic acid (**1**) is the dominant component in three PF plants, and no significant variance of its content is observed among the three species. However, compounds **2**–**5** are more abundant in *P. sheareri* (Great Shiwei) than *P. lingua* or *P. petiolosa* (Little Shiwei), which is a potential chemical characteristic to distinguish between Great Shiwei and Little Shiwei. Furthermore, the absence of trifolin (**5**) in *P. petiolosa* indicates a significant difference between *P. petiolosa* and the other two species. All above results are consistent with the fact that the three species show some difference in therapeutic effect in clinical application.

Multivariate analysis was also implemented after pattern analysis and monitoring. As a result, each compound pattern of three species was identified, making it easy to distinguish between the three.

In future research, it will be necessary to validate this method by combining it with more comprehensive pharmacological comparison among different species. The five key chemicals **1**–**5** found in PF could be further investigated in vitro and in vivo to identify their biological contributions to the variations in clinic effect among the three species.

This is the first report on the simultaneous determination of five main compounds in PF. The developed method was proved to be simple, rapid, accurate, and reliable. Under the multiple optimized HPLC conditions, five compounds were totally separated and eluted individually within 40 min. The validation was employed to analyze 35 PF samples of different plant and geographic origins. Results indicated that although the chemical constituents of samples from different plant origins or geographic regions were similar, the content of each compound varied greatly. Based on the HPLC data of 35 collections, PCA and PLS-DA can be used to clearly visualize the correlation and discrimination between samples from three species. In conclusion, the established method is reliable, rapid, sensitive, and integrative for the quality assessment and discrimination of Pyrrosiae Folium samples derived from different plant species and geographic origins.

## 4. Materials and Methods

### 4.1. Reagents and Materials

Acetonitrile and formic acid were of HPLC grade (Burdick and Jackson, Honeywell International Inc., Muskegon, MI, USA). Water used in HPLC analysis was prepared using a Milli-Q Water purification system (Millipore, MA, USA). Reference standards, chlorogenicacid, mangiferin, isomangiferin, and astragalin were purchased from Chengdu Must Bio-Technology Co., Ltd. (Chengdu, China), while trifolin was from Shanghai Tanto Biotech Co., Ltd. (Shanghai, China). Their purity was verified to be more than 98% by HPLC analysis. The samples of Pyrrosiae Folium were collected from different geographic areas and were authenticated as the leaves of *Pyrrosia sheareri* (Bak.) Ching, *P. lingua* (Thunb.) Farwell, and *P. petiolosa* (Christ) Ching (Figure 1), respectively, by Dr. Sibao Chen based on herbarium specimen (deposited in the Herbarium of State Key Laboratory of Chinese Medicine and Molecular Pharmacology, Shenzhen, China).

### 4.2. Instrumentation and Liquid Chromatography

HPLC performance was conducted using an Agilent 1200 liquid chromatography system (Agilent Technologies, Palo Alto, CA, USA), equipped with a quaternary solvent deliver system, an auto-sampler, and a DAD detector. A Grace VisionHT C_18_–HL column (250 mm × 4.6 mm, 5 μm) at an ambient temperature of about 25 °C was applied for all analyses. Detection wavelength was set at 254 nm. The mobile phase consists of (A) acetonitrile and (B) 0.05% formic acid aqueous solution (*v*/*v*), and the elution program was performed by using a gradient elution of 9–12% A at 0–5 min, 12–14% A at 5–15 min, 14–17% A at 15–25 min, 17–18% A at 25–40 min. The flow rate was 1.0 mL/min and aliquots of 10 μL were injected.

### 4.3. Preparation of Standard and Sample Solutions

The mixture standard stock solution was prepared by dissolving the reference substances in 50% methanol to a final concentration of 289.4 μg/mL of chlorogenic acid, 116.4 μg/mL of mangiferin, 228.8 μg/mL of isomangiferin, 95.9 μg/mL of trifolin, and 98.6 μg/mL of astragalin, respectively. Accurately weighed 0.2 g of powder of dried samples was ultra-sonicated with 10 mL of 50% methanol for 30 min under ambient temperature. This extraction was repeated twice. The extractive was combined and filtrated through analytical filter paper and then the filtered solution was evaporated to dryness in vacuum. The dry extract was dissolved in 10 mL of 50% methanol and filtrated through a 0.45-μm membrane filter. Then, 10 μL of each sample solution was analyzed by HPLC. The contents of the analytes were determined from the corresponding calibration curves.

### 4.4. Method Validation

The mixture stock solution was diluted into a serial of appropriate concentrations for calibration curve establishment. The linearity of each analyte was determined with three injections for six concentrations and plotted using the linear regression of the mean peak area versus concentration. The limit of detection (LOD) and quantification (LOQ) under the chromatographic conditions were determined by measuring the Signal-to-Noise ratio (S/N) for each compound by injecting a series of solutions until the S/N ratio was equal to 3 for LOD and 10 for LOQ, respectively.

Intra- and inter-day variations were detected for the precision evaluation of the method. The intra-day variation was examined by determining the contents of five analytes in one sample (batch 1) in six replicates during a single day, and inter-day variation was determined by duplicating the experiments on three successive days, respectively. Variations were expressed as the relative standard deviations (RSD).

The recovery was used to evaluate the accuracy of the method. Known quantities of the mixed standard solution were added into the known amounts of sample (batch 1), and then the mixed samples were extracted and analyzed with the above-described method. The added standard solution was prepared in the concentration ranges of calibration curve with three different concentration levels (high, middle, and low), and triplicate experiments were performed at each level. The recovery was calculated as follow: Recovery (%) = 100 × amount detected / amount spiked.

Stability was tested with mixture stock solution and sample solution that were stored at 20–25 °C and analyzed every 12 h over 3 days. The relative standard deviation was taken as a measure of stability.

### 4.5. Multivariate Statistic Analysis

PCA and PLS-DA were carried out based on the content of five bioactive markers in 35 samples of PF representing different geographical regions of China, using STATISTICA 7.0 software (TIBCO Software Inc., Palo Alto, CA, USA). As for PCA, the first three principle components accounted for more than 90% of variance. As for PLS-DA, the data matrix performed a variable reduction on the dataset by calculating new variables (called latent components or factors) combining the variables in the dataset in order to find the maximum correlation between them and the class variable and, thus, the maximum separation among the classes. For an acceptance model, a high percentage of correct classification should be obtained.

## Figures and Tables

**Table 1:** Linear relation between peak area and concentration.

Analytes	Regression Equation	*r*^2^	Linear Range (μg/mL)	LOD (μg/mL)	LOQ (μg/mL)
Chlorogenic acid	*y* = 20.427*x* − 20.31	0.9995	2.89–289.4	0.053	0.178
Mangiferin	*y* = 81.058*x* − 38.27	0.9996	1.16–116.4	0.014	0.047
Isomangiferin	*y* = 61.572*x* − 46.64	0.9997	2.29–228.8	0.030	0.101
Trifolin	*y* = 32.979*x* − 16.29	0.9996	0.96–95.9	0.085	0.284
Astragalin	*y* = 42.194*x* − 18.94	0.9996	0.99–98.6	0.047	0.155

In the regression equation *y* = a*x* + b, *x* refers to the concentration of analytes (μg/mL); *y* is the peak area; and *r*^2^ is the correlation coefficient of the equation; LOD is the limit of detection; LOQ is the limit of quantification.

**Table 2:** Intra-day and inter-day variations of analytes in Pyrrosiae Folium sample (Batch 1).

Analyte	Inter-Day (*n* = 3)							
	Day 1		Day 2		Day 3		Mean ± SD ^a^	RSD (%)
	Mean ± SD ^a^	RSD (%)	Mean ± SD ^a^	RSD (%)	Mean ± SD ^a^	RSD (%)		
**1**	4426.51 ± 43.32	0.98	4453.84 ± 50.76	1.14	4471.57 ± 34.61	0.77	4450.64 ± 45.01	1.01
**2**	165.53 ± 1.49	0.90	167.74 ± 2.37	1.41	168.41 ± 1.45	0.86	167.23 ± 2.59	1.27
**3**	421.83 ± 5.39	1.28	424.56 ± 4.18	0.99	424.45 ± 2.18	0.51	423.61 ± 4.10	0.97
**4**	189.11 ± 4.56	2.40	182.85 ± 3.78	2.06	183.91 ± 3.42	1.85	185.29 ± 4.66	2.50
**5**	246.56 ± 5.69	2.31	244.02 ± 5.78	2.37	241.47 ± 5.72	2.37	244.02 ± 5.79	2.37

^a^ Data are microgram compounds per gram crude drug.

**Table 3:** Recovery of analytes in Pyrrosiae Folium (*n* = 3).

Analyte	Amount Spiked (μg/mL)	Amount Detected ^a^ (μg/mL)	Recovery ^b^ (%)	RSD (%)
	5.79	5.84	100.95	2.90
**1**	11.58	12.04	104.00	2.99
	115.76	114.14	98.60	2.30
	2.33	2.34	100.66	0.72
**2**	4.66	4.58	98.33	2.41
	46.56	47.85	102.78	1.62
	4.58	4.70	102.93	2.61
**3**	9.15	9.11	99.58	0.87
	91.52	92.04	100.57	1.90
	1.92	1.89	100.56	3.48
**4**	3.84	3.89	101.41	3.44
	38.35	38.60	100.66	1.65
	1.97	1.93	97.76	4.21
**5**	3.94	3.82	97.35	2.27
	39.44	36.74	93.16	1.80

^a^ Calculated as total amount found−total original amount. Data are the means of three experiments; ^b^ Calculated as 100% × (detected amount/added amount). Data are the means of three experiments.

**Table 4:** Stability of mixture stock solution and sample solution stored at 20–25 °C for 3 days.

Analyte	Mixture Stock Solution		Sample Solution	(Batch 1)
	Mean ± SD ^a^	RSD (%)	Mean ± SD ^b^	RSD (%)
**1**	113.62 ± 1.22	1.07	4478.43 ± 37.56	0.84
**2**	45.62 ± 0.65	1.42	167.53 ± 2.13	1.27
**3**	89.97 ± 0.92	1.02	425.11 ± 4.14	0.97
**4**	37.41 ± 0.61	1.63	186.48 ± 3.21	1.71
**5**	36.82 ± 0.58	1.57	245.12 ± 3.54	1.45

^a^ Data are microgram compounds per milliliter; ^b^ Data are microgram compounds per gram crude drug.

**Table 5:** Contents of compounds **1**–**5** in Pyrrosiae Folium samples (*n* = 3).

Species	Sample No.	Contents of Compounds 1–5 (μg/g) ^a^				
		1	2	3	4	5
*P. sheareri*	1	4349.03	170.03	421.61	184.29	237.55
	2	4070.53	5400.74	10,916.67	108.47	275.21
	3	3895.50	358.32	1026.59	103.93	176.88
	4	3760.14	427.28	1334.93	103.27	198.27
	5	2190.22	320.00	916.24	106.03	338.69
	6	5187.23	361.85	986.24	114.78	332.41
	7	4493.57	390.55	1017.95	121.04	219.57
	8	4931.57	434.99	1118.37	121.30	209.62
	9	4104.04	406.10	1148.48	110.96	202.33
	10	3549.57	259.43	770.29	118.30	101.60
	Mean	4053.14	852.93	1965.74	119.24	229.23
*P. lingua*	11	5802.53	93.37	297.25	114.03	89.40
	12	7277.73	57.39	72.84	526.78	ND ^b^
	13	8957.35	58.91	126.41	1097.87	117.89
	14	9780.12	57.32	103.14	943.82	ND
	15	9672.51	57.52	93.65	889.28	ND
	16	8089.00	99.35	121.52	571.26	ND
	17	1507.21	ND	82.32	141.60	ND
	18	4308.91	ND	164.21	125.48	120.16
	19	8935.14	52.87	123.71	1194.43	113.58
	20	5652.18	ND	185.12	100.61	72.17
	Mean	6998.27	68.10	137.02	570.52	102.64
*P. petiolosa*	21	8424.82	84.06	97.19	691.07	ND
	22	3753.03	ND	145.33	93.06	ND
	23	3634.47	ND	152.79	76.35	ND
	24	4248.43	ND	153.01	ND	ND
	25	4561.98	ND	121.86	ND	ND
	26	3748.44	ND	117.55	89.88	ND
	27	10990.55	121.23	98.77	1012.30	ND
	28	9693.31	86.58	94.23	1043.10	ND
	29	11,448.08	121.55	101.64	1163.98	ND
	30	11,172.10	129.29	ND	1069.24	ND
	31	9253.95	76.66	87.09	784.87	ND
	32	10,069.68	107.30	92.70	1032.65	ND
	33	7440.68	83.13	ND	1015.50	ND
	34	9464.51	114.53	ND	943.21	ND
	35	8012.01	73.75	86.57	654.62	ND
	Mean	7727.74	99.81	112.39	743.83	ND

^a^ Data are expressed as the means of three tests; ^b^ Under detectable level.
